# Increased Short-Term Beat-to-Beat QT Interval Variability in Patients with Impaired Glucose Tolerance

**DOI:** 10.3389/fendo.2017.00129

**Published:** 2017-06-13

**Authors:** Andrea Orosz, István Baczkó, Szabolcs Nyiraty, Anna E. Körei, Zsuzsanna Putz, Róbert Takács, Attila Nemes, Tamás T. Várkonyi, László Balogh, György Ábrahám, Péter Kempler, Julius Gy. Papp, András Varró, Csaba Lengyel

**Affiliations:** ^1^Department of Pharmacology and Pharmacotherapy, University of Szeged, Szeged, Hungary; ^2^First Department of Medicine, University of Szeged, Szeged, Hungary; ^3^First Department of Medicine, Semmelweis University, Budapest, Hungary; ^4^Second Department of Medicine and Cardiology Centre, University of Szeged, Szeged, Hungary; ^5^Juhász Gyula Faculty of Education, Institute of Physical Education and Sport Science, University of Szeged, Szeged, Hungary; ^6^MTA-SZTE Research Group of Cardiovascular Pharmacology, Hungarian Academy of Sciences, Szeged, Hungary

**Keywords:** cardiovascular autonomic neuropathy, impaired glucose tolerance, prediabetes, proarrhythmic risk, short-term variability of the QT interval, sudden cardiac death, QT dispersion, QT prolongation

## Abstract

Prediabetic states and diabetes are important risk factors for cardiovascular morbidity and mortality. Determination of short-term QT interval variability (STV_QT_) is a non-invasive method for assessment of proarrhythmic risk. The aim of the study was to evaluate the STV_QT_ in patients with impaired glucose tolerance (IGT). 18 IGT patients [age: 63 ± 11 years, body mass index (BMI): 31 ± 6 kg/m^2^, fasting glucose: 6.0 ± 0.4 mmol/l, 120 min postload glucose: 9.0 ± 1.0 mmol/l, hemoglobin A1c (HbA1c): 5.9 ± 0.4%; mean ± SD] and 18 healthy controls (age: 56 ± 9 years, BMI: 27 ± 5 kg/m^2^, fasting glucose: 5.2 ± 0.4 mmol/l, 120 min postload glucose: 5.5 ± 1.3 mmol/l, HbA1c: 5.4 ± 0.3%) were enrolled into the study. ECGs were recorded, processed, and analyzed off-line. The RR and QT intervals were expressed as the average of 30 consecutive beats, the temporal instability of beat-to-beat repolarization was characterized by calculating STV_QT_ as follows: STV_QT_ = Σ|QT_n + 1_ − QT_n_| (30x√2)^−1^. Autonomic function was assessed by means of standard cardiovascular reflex tests. There were no differences between IGT and control groups in QT (411 ± 43 vs 402 ± 39 ms) and QTc (431 ± 25 vs 424 ± 19 ms) intervals or QT dispersion (44 ± 13 vs 42 ± 17 ms). However, STV_QT_ was significantly higher in IGT patients (5.0 ± 0.7 vs 3.7 ± 0.7, *P* < 0.0001). The elevated temporal STV_QT_ in patients with IGT may be an early indicator of increased instability of cardiac repolarization during prediabetic conditions.

## Introduction

Prediabetic states and diabetes are important risk factors for cardiovascular morbidity and mortality (1–3). Cardiovascular death or death of unknown origin was in the 0.4–0.5% range in the subgroups of a 3-year follow-up study on patients with impaired glucose tolerance (IGT) and/or impaired fasting glucose (IFG) level (4). In a 23-year follow-up study on Japanese American men, the relative risks for sudden cardiac death were 2.22 in subjects with asymptomatic hyperglycemia, and 2.76 in diabetic patients (5). Diabetes status was a strong risk factor for sudden death, but not for fatal myocardial infarction in men during the population-based Paris Prospective Study I (6). Higher risk of sudden cardiac death was associated with borderline diabetes, diabetes with or without microvascular disease, compared to subjects without diabetes in a population-based case–control study of patients experienced out-of-hospital cardiac arrest due to heart disease (1). Out-of-hospital sudden cardiac deaths were 1.79-fold for non-diabetic men with impaired fasting plasma glucose and 2.26-fold for men with type 2 diabetes, and a 1 mmol/l increment in fasting plasma glucose was related to an increase of 10% in the risk of sudden cardiac death in Finnish men (7).

The prevalence of confirmed cardiovascular autonomic neuropathy (AN), an impairment of autonomic control of the cardiovascular system during diabetes, was between 16 and 20% in unselected type 1 and type 2 diabetic patients (8). Cardiovascular AN is a risk marker of cardiovascular morbidity, and it causes a 3.65-fold increase in the relative risk of mortality (8). Cardiac AN promotes ventricular repolarization disturbances [QTc prolongation, increased QT dispersion (QTd)] and may increase the risk of sudden cardiac death. Prolongation of QT interval could lead to increased myocardial electrical instability, predisposing diabetic subjects with AN to potentially fatal ventricular arrhythmias (9). Cardiac AN with QT interval prolongation proved to be a poor prognostic factor for sudden cardiac death in diabetic patients in a 5-year follow-up study (10). Prolonged QTc is more frequent in patients with IFG (30%) and with diabetes (42%) than in subjects with normal glucose tolerance (22%), and both IFG and diabetes increased the risk of prolonged QTc (11). QTc interval duration was found to be significantly higher both during the day and night using ECG Holter recordings in patients with IGT compared to subjects with normal glucose tolerance (12). IGT was confirmed in 15% of men and 23% of women with QTc prolongation (>440 ms) in the population-based Hisayama study in Japan (13).

In the clinical setting, the risk assessment of serious ventricular arrhythmias in individual patients is challenging since the prolongation of repolarization that manifests as QT interval prolongation on the ECG does not always correlate with subsequent development of ventricular arrhythmias (14–16). Cardiac repolarization reserve may be reduced even without significant changes in the duration of cardiac repolarization; therefore, QT interval prolongation cannot reliably predict the development of ventricular arrhythmias (17). The short-term variability of the QT interval (STV_QT_) was introduced as an early and sensitive indicator of repolarization instability (18) that more reliably predicted ventricular arrhythmias and sudden cardiac death than prolongation of repolarization in previous experimental (16, 19–22) and clinical studies (23–27). Type 1 diabetes mellitus moderately lengthened ventricular repolarization, attenuated repolarization reserve, and enhanced the risk of sudden cardiac death in dogs (27, 28), and similar mechanisms might also occur in patients suffering from prediabetic states and diabetes.

The aim of the present study was to determine beat-to-beat STV_QT_ for assessment of repolarization instability and possible proarrhythmic risk, together with cardiovascular autonomic function in patients with IGT.

## Materials and Methods

### Patient Population

Patients with IGT who are followed at the First Department of Medicine, Semmelweis University, Budapest, Hungary, were eligible for this study. Patients were excluded if they had excessive (>5%) ectopic atrial or ventricular beats, were in a rhythm other than normal sinus, had repolarization abnormalities (i.e., early repolarization pattern, T wave inversion, and complete left bundle branch block or right bundle branch block), had a permanent pacemaker or any other disorders such as serious retinopathy, symptomatic cardiac and pulmonary disease, and acute metabolic disease, had excessive noise on the electrocardiographic signal that precluded analysis of the ECG waveform, were on any medication likely to affect the investigated ECG parameters, or consumed significant amount of food within 3 h or drank alcohol, coffee, or smoked within 10 h.

We studied 18 IGT patients, 9 males and 9 females with the age of 63 ± 11 years (all values presented are mean ± SD). A total of 18 age- and sex-matched volunteers (mean age 56 ± 9 years) without a history or evidence of heart disease were enrolled in the study as controls. All of the control individuals and IGT patients were of Caucasian origin.

The studies described here were carried out in accordance with the Declaration of Helsinki (2000) of the World Medical Association and were approved by the Scientific and Research Ethical Committee of the Medical Research Council at the Hungarian Ministry of Health (ETT-TUKEB), under ethical approval No. 4987-0/2010-1018EKU (338/PI/010). All subjects have given written informed consent of the study.

### Data Collection and Analysis

Before the ECG recording, all IGT patients and controls were at rest, in the supine position for 10 min. Then, 12-lead electrocardiograms were continuously recorded for 5 min at rest, also in the supine position to obtain signals with the least amount of motion artifact. In all leads, the ECG signals were digitized at 2,000 Hz sampling rate with a multichannel data acquisition system (Cardiosys-A01 software, MDE Heidelberg GMBH, Heidelberg, Germany) connected to a personal computer and stored for later off-line analysis.

Out of the repolarization parameters, we analyzed the frequency corrected QT interval (QTc) using Bazett’s (QTc = QT/√RR), Fridericia (QTc = QT/[RR/1,000]1/3), Framingham (QTc = QT + [0.154 × {1,000 − RR}]) and the Hodges formulas (QTc = QT + 1.75 × [60,000/RR − 60]), the QTd, the PQ and QRS intervals, the duration of terminal part of T waves (T_peak_ − T_end_) and the short-term variability of QT interval (STV_QT_).

The RR and QT intervals, as well as duration of the T wave from the peak to the end (T_peak_ − T_end_) intervals were measured semi-automatically in 30 consecutive beats (minimum number of intervals needed for variability measurements) and were calculated as the average of 30 beats. The QT intervals were analyzed by conventional computerized QT measurement technique, all QT intervals were checked in a blinded manner by the same expert investigator of the team and fiducial cursor positions set by the software were manually corrected if needed (29). QTc interval duration was defined as the mean duration of all QTc intervals measured. The PQ and QRS intervals were measured as the average of 15 consecutive beats. All measurements were carried out using limb lead II and in case of excessive noise in limb lead II, lead V5.

To characterize the temporal instability of beat-to-beat heart rate (HR) and repolarization, Poincaré plots of the QT and RR intervals were constructed, where each QT and RR value is plotted against its former value. STV_QT_ and STV_RR_ were calculated using the following formula: STV = Σ|D_n+1_ − D_n_| (30x√2)^−1^, where *D* represents the duration of the QT and RR intervals. This calculation defines the STV as the mean distance of points perpendicular to the line of identity in the Poincaré plot and relies on previous mathematical analysis (30).

Autonomic function was assessed by means of five standard cardiovascular reflex tests: the HR responses to deep breathing and to standing up (30/15 ratio), the Valsalva maneuver, the systolic blood pressure response to standing up, and the diastolic pressure change during a sustained handgrip (31). A score was created to express the severity of AN, based on the results of the five tests (normal: 0, borderline: 1, abnormal: 2). The total score was in the interval of 0–10.

Fasting venous blood samples were obtained from each patient and controls for the determination of serum glucose and hemoglobin A1c (HbA1c) levels. Oral glucose tolerance test (OGTT) was carried out with 75 g glucose to confirm the diagnosis of IGT according to the World Health Organization recommendation (120 min value in 7.8–11.0 mmol/l range).

### Statistical Analysis

All data are expressed as mean ± SD. Comparisons between IGT patients and controls for the study variables were done using the unpaired Student’s *t*-test for normally distributed parameters (D’Agostino-Pearson test was used to assess normality of distribution), and linear regression for revealing correlations. The statistical analyses were performed using the Statistica 12 software package. Statistical significance was defined by *P* < 0.05 level.

## Results

### Clinical Data of IGT Patients and Control Subjects

In 18 IGT patients studied, mean body mass index (BMI) was significantly higher (*P* < 0.05) than among age- and sex-matched healthy volunteers. Mean systolic blood pressure did not differ significantly between control subjects and IGT patients; however, IGT patients had lower diastolic blood pressure (74 ± 9 vs 81 ± 10 mmHg; *P* < 0.05). Significant differences were seen between IGT and control groups in mean serum glucose (6.0 ± 0.4 vs 5.2 ± 0.4 mmol/l; *P* < 0.0001), HbA1c (5.9 ± 0.4 vs 5.4 ± 0.3%; *P* < 0.0001), and serum glucose 120 min level during OGTT (9.0 ± 1.0 vs 5.5 ± 1.3 mmol/l; *P* < 0.0001). Clinical data of IGT patients and control subjects are shown in Table 1.

**Table 1:** Clinical data of IGT patients and age-matched control subjects.

	Control	Patients with IGT
*n*	18	18
Sex (male/female)	9/9	9/9
Age (year)	56 ± 9	63 ± 11
Weight (kg)	79 ± 19	88 ± 17
Height (cm)	170 ± 11	168 ± 6
BMI (kg/m^2^)	27 ± 5	31 ± 6*
Systolic BP (mmHg)	130 ± 12	134 ± 17
Diastolic BP (mmHg)	81 ± 10	74 ± 9*
0 min glucose (mmol/l)	5.2 ± 0.4	6.0 ± 0.4**
120 min glucose (mmol/l)	5.5 ± 1.3	9.0 ± 1.0**
HbA1c (%)	5.4 ± 0.3	5.9 ± 0.4**

Values are represented as mean ± SD. Values are considered statistically significantly different at *P* < 0.05 (*), *P* < 0.0001 (**) compared with the control group.

*IGT, impaired glucose tolerance; BMI, body mass index; BP, blood pressure; HbA1c, hemoglobin A1c*.

### Electrocardiographic Parameters in Study Subjects

Comparison of the two groups (IGT patients vs controls) revealed no significant differences in HR, the PQ, QRS, QT and T_peak_ − T_end_ intervals and the QTd. In order to reliably assess the duration of ventricular repolarization and to minimize the influence of changing HR on the QT interval, the frequency corrected QT interval (QTc) was calculated by the Bazett’s, Fridericia, Framingham and Hodges formulas. QTc values calculated with all the four formulas showed no significant differences between IGT patients and controls. Electrocardiographic parameters in study subjects are presented in Table 2.

**Table 2:** Electrocardiographic parameters in patients with IGT and age-matched controls.

	Control	Patients with IGT
RR (ms)	900 ± 144	914 ± 163
PQ (ms)	161 ± 18	162 ± 24
QRS (ms)	94 ± 9	94 ± 8
QT (ms)	402 ± 39	411 ± 43
QTc (ms) Bazett	424 ± 19	431 ± 25
QTc (ms) Fridericia	416 ± 23	424 ± 27
QTc (ms) Framingham	417 ± 22	424 ± 26
QTc (ms) Hodges	416 ± 25	424 ± 29
QTd (ms)	42 ± 17	44 ± 13
T_peak_ − T_end_ (ms)	86 ± 14	88 ± 23
T wave amplitude (μV)	220 ± 119	225 ± 120
STV_RR_ (ms)	18.5 ± 14.3	10.5 ± 6.7*
STV_QT_ (ms)	3.7 ± 0.7	5.0 ± 0.7**

Values are represented as mean ± SD. Values are considered statistically significantly different at *P* < 0.05 (*), *P* < 0.0001 (**) compared with the control group. *n* = 18 in each group.

*IGT, impaired glucose tolerance; QTc, frequency corrected QT interval (calculated by the Bazett’s, Fridericia, Framingham and Hodges formulas); QTd, QT dispersion; T_peak_ − T_end_, duration of the T wave from the peak to the end; STV_RR_, beat-to-beat short-term temporal variability of the RR interval; STV_QT_, beat-to-beat short-term temporal variability of the QT interval*.

### Short-term Beat-to-Beat Variability of the QT and RR Intervals

As it has been shown that T wave amplitude may affect STV_QT_ (32), we have also compared the T wave amplitudes in both groups. T wave amplitudes did not differ significantly between IGT patients and control subjects (225 ± 120 vs 220 ± 119 μV, *P* = 0.882).

To characterize the instability of cardiac ventricular repolarization, the short-term beat-to-beat variability of the QT interval was calculated in IGT patients and age-matched controls. Since it is reasonable to assume that STV_QT_ can be, at least in part, influenced by the short-term variability of the RR interval, the STV_RR_ was also calculated in both groups (33). Patients with IGT exhibited a significantly lower STV_RR_ compared to controls (10.5 ± 6.7 vs 18.5 ± 14.3 ms, *P* = 0.0373). No significant correlation was found between STV_QT_ and STV_RR_ values in IGT patients (*r* = −0.3152; *P* = 0.203).

**Figure 1:**
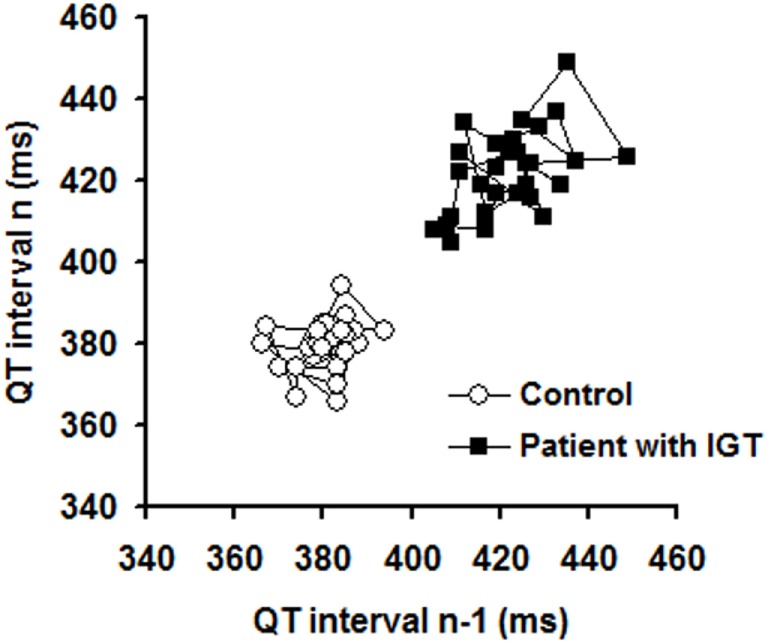
Representative Poincaré plots illustrating short-term temporal variability of the QT interval in a control individual and in a patient with impaired glucose tolerance (IGT).

As individual representative examples (Poincaré plots) illustrate (Figure 1) and grouped average data show (Table 2), STV_QT_ was significantly increased by 36% in IGT patients compared to controls (5.0 ± 0.7 ms vs 3.7 ± 0.7 ms, *P* < 0.0001).

### Cardiovascular Autonomic Function

Standard cardiovascular reflex tests indicated significant deteriorations in Valsalva ratio (*P* < 0.0001) and the HR responses to deep breathing among IGT subjects compared to controls (*P* = 0.033). However, no significant differences in 30/15 ratio, systolic blood pressure response after standing up, diastolic blood pressure response after sustained handgrip, and AN score were detected between the two groups. Autonomic parameters of IGT patients and age-matched control subjects are shown in Table 3.

**Table 3:** AN parameters of IGT patients and age-matched control subjects.

	Control	Patients with IGT
Heart rate (HR) variation during deep breathing (1/min)	16 ± 7	11 ± 8*
Valsalva ratio	1.7 ± 0.3	1.2 ± 0.1**
30/15 ratio	1.3 ± 0.3	1.2 ± 0.1
Systolic BP fall after standing up (mmHg)	8 ± 8	6 ± 7
Diastolic BP increase after sustained handgrip (mmHg)	11 ± 6	14 ± 6
AN score	2.4 ± 1.2	2.7 ± 1.3

Values are represented as mean ± SD. Values are considered statistically significantly different at *P* < 0.05 (*), *P* < 0.0001 (**) compared with the control group. *n* = 18 in each group.

*IGT, impaired glucose tolerance; 30/15 ratio, immediate HR response to standing; BP, blood pressure; AN, autonomic neuropathy*.

### Correlation of Short-term QT Interval Variability (STV_QT_) with Laboratory Data and AN Parameters in Patients with IGT

Pearson correlation coefficient values indicated that neither laboratory data nor autonomic parameters correlated with STV_QT_, these data are presented in Table 4. However, 30/15 ratio had significant negative correlation with STV_QT_ (*r* = –0.4729; *P* = 0.048).

**Table 4:** Correlation of short-term QT interval variability (STV_QT_) with laboratory data and AN parameters in patients with IGT.

	STV_QT_ in patients with IGT (ms)	
	Pearson *r*	*P* value (two-tailed)
HbA1c (%)	0.2708	0.277
OGTT 0 min (mmol/l)	0.2118	0.399
OGTT 120 min (mmol/l)	−0.1118	0.659
Heart rate (HR) variation during deep breathing (1/min)	−0.0379	0.881
Valsalva ratio	0.1101	0.664
30/15 ratio	−0.4729	0.048*
Systolic BP fall after standing up (mmHg)	−0.0163	0.949
Diastolic BP increase after sustained handgrip (mmHg)	−0.0685	0.787
AN score	−0.1353	0.593

Values are represented as Pearson correlation coefficient. Values are considered statistically significantly different at *P* < 0.05 (*).

*STV_QT_, beat-to-beat short-term temporal variability of the QT interval; IGT, impaired glucose tolerance; HbA1c, hemoglobin A1c; OGTT, oral glucose tolerance test; 30/15 ratio, immediate HR response to standing; BP, blood pressure; AN, autonomic neuropathy*.

## Discussion

Cardiac autonomic dysfunction present in prediabetes may lead to repolarization disturbances and may increase the risk of ventricular arrhythmias and sudden cardiac death. In this study, we show for the first time that beat-to-beat STV_QT_, an early and sensitive parameter of repolarization instability, is increased even before QTc prolongation or enhanced QTd could be detected in patients with IGT.

Patients with prediabetic conditions or diabetes have higher risk for sudden cardiovascular death (1, 5–7). Cardiac AN and instability of cardiac repolarization, detected by QTc prolongation or increased QTd, contribute to the increased risk for sudden cardiac death (9, 10). Prolonged QTc was related to a progressive worsening of glucose tolerance after adjustment for possible confounding factors in elderly women with IGT or diabetes (34). Impairment of cardiac parasympathetic and sympathetic innervation as well as QT interval prolongation may play a partial role in the pathogenic mechanism of sudden unexpected death in diabetic patients. Cardiovascular adaptation mechanisms, including baroreflex sensitivity and HR variability, are also impaired in diabetic AN that may further increase the risk for arrhythmia development (35).

However, decreased repolarization capacity and increased arrhythmia susceptibility is not necessarily preceded by significant changes in the duration of cardiac repolarization, and in these cases, cardiac repolarization reserve may be reduced without manifest QT interval prolongation (17). Importantly, a wide range of non-cardiovascular drugs or even dietary constituents with only mild repolarization blocking effects can increase the risk for serious ventricular arrhythmias and sudden cardiac death in patients with impaired repolarization reserve (17). Therefore, in this clinical setting, the prediction of lethal ventricular arrhythmias is especially challenging. STV_QT_ has been suggested as an early and sensitive indicator of temporal repolarization instability based on previous experimental and clinical studies (16, 18, 20, 24–26).

Our present study is the first to indicate that patients with IGT, a prediabetic condition, have repolarization instability indicated by elevated beat-to-beat STV_QT_. This study was not designed to assess the exact mechanisms responsible for repolarization disturbances in patients with IGT; however, several possible mechanisms may be considered. Compelling recent evidence suggests a direct link between type 2 ryanodine receptor (RyR2) dysfunction in the endo/sarcoplasmic reticulum leading to altered intracellular calcium homeostasis, glucose intolerance, and impaired insulin secretion in patients with catecholaminergic polymorphic ventricular tachycardia (CPVT) (36, 37). The known *RYR2* mutations identified in these CPVT patients were previously linked to reduced binding affinity of calstabin2 to the RyR2 channel resulting in intracellular Ca^2+^ leak (37–39). In knock-in mouse models where these CPVT-linked mutations leading to RyR2-mediated Ca^2+^ leak were reconstituted, mitochondrial dysfunction and blunted ATP production with concomitantly increased sarcolemmal K_ATP_ channel function (reversible by the K_ATP_ blocker glibenclamide) were found in pancreatic β-cells to cause reduced insulin secretion and consequently, IGT (36). In addition to causing altered glucose metabolism and providing triggers for cardiac arrhythmias (CPVT), the RyR2-mediated Ca^2+^ leak—by depleting Ca^2+^ stores—may also contribute to arrhythmia substrate creation *via* reduced I_Ks_ current, i.e., decreased Ca^2+^-dependent I_Ks_ activation (40) and consequently, impaired repolarization reserve (17). Interestingly, and in accordance with this mechanism, reduced I_Ks_ density, impaired repolarization reserve, and increased risk for sudden cardiac death were described in diabetic dogs (28). Although there is no doubt that RyR2 channel dysfunction is directly linked to heart failure (41), cardiac arrhythmia development (42, 43), IGT, and reduced insulin release (36, 44), however, further clinical studies are needed to determine whether *RYR2* mutations leading to leaky RyR2 channels are frequently present in patients diagnosed with IGT in general.

Repolarization instability can be a long-standing risk factor for cardiovascular morbidity and mortality in prediabetic states and during development of diabetes. However, the role of additional cardiovascular risk factors cannot be excluded in early prediabetic conditions. Early sympathetic nerve dysfunction and insulin resistance may also play a role in the development of decreased coronary flow reserve in patients with normoglycemia (45). In this regard, increased QT interval variability associated with sympathetic dysinnervation was observed in patients with type 2 diabetes in the supine position and the QT variability was further elevated in the context of sympathetic activation upon standing (46).

Relative sympathetic predominance was observed in cardiovascular reflex tests during IGT, as sympathetic parameters (systolic BP fall after standing up and diastolic BP increase after sustained handgrip) were unchanged, whereas two of three parasympathetic parameters measured (HR variation and Valsalva ratio) were significantly decreased. In addition, a significant negative correlation was seen between the values measured in the third parasympathetic test (30/15 ratio) and STV_QT_ in our study. The significantly lower STV_RR_ values observed also represent this parasympathetic dysinnervation and subsequent relative sympathetic predominance in patients with IGT. Sympathetic predominance acutely evoked by graded head-up tilt test resulted in similar changes, such as decreased variance of HR and increased variance of repolarization duration (47, 48).

The prevalence of distal symmetric polyneuropathy that may result in weakness, sensory loss, pain, autonomic dysfunction, gait impairment, falls, and disability has been reported to be 11% in patients with IGT (49). It is known that IGT is present in about 40% of patients with idiopathic peripheral neuropathy and abnormal microvascular endothelial dysfunction is common in both patient groups (50). It has long been known that IGT is associated with AN and a shift is observed in sympathovagal balance to sympathetic overactivity (51–54). Prevalence of parasympathetic dysfunction was 25%, whereas the prevalence of sympathetic dysfunction was 6% in 268 patients with IGT in the Finnish Diabetes Prevention Study (55). Abnormal sinus arrhythmia test (55 vs 33%; *P* = 0.004) and abnormal Valsalva maneuver (34 vs 7%; *P* = 0.004) were significantly more frequent in patients with IGT than in control subjects; however, the frequency of abnormal postural test was not different in these two groups (*P* = 0.334) (51). Insulin resistance was associated with global autonomic dysfunction and an increased LF/HF (low frequency/high frequency) ratio indicating sympathetic overactivity (52). However, the autonomic dysfunction was less significant in IGT patients than in diabetic subjects (52). IGT induced decrease in parasympathetic modulation (decreased HF power and 30/15 ratio) and a shift toward augmented sympathetic tone (increased LF/HF ratio) were also confirmed in an epidemiological study (54).

Putz et al. (53) described a mainly subclinical, asymptomatic small-fiber neuropathy, and mild impairment of cardiovascular autonomic function in IGT subjects. Similar to our present findings, HR variation and Valsalva ratio were decreased, whereas 30:15 ratio was unchanged among the tests evaluating parasympathetic activity; however, sympathetic function was also mildly impaired in patients with IGT (53). Moreover, these IGT patients also have abnormal circadian blood pressure regulation and increased diastolic blood pressure (56). Abnormal HR recovery was more common in patients with IFG (42%) and diabetes (50%) than in participants with normal glucose tolerance (31%) in a population-based Italian study; the relative risks were 1.34 (95% confidence intervals = 1.2–1.5) and 1.61 (95% CI = 1.35–1.92), respectively (57).

Fasting plasma glucose found to be an independent predictor of abnormal HR recovery (*P* < 0.0003) even after adjustments for other confounders (57). Moreover, impaired glucose regulation significantly (*P* < 0.006) correlated with adrenergic autonomic dysfunction when age, an important confounder, was removed from the model (58). The self-assessment of autonomic symptoms by patients with IGT and early diabetes correlated to the degree of autonomic dysfunction defined by abnormal 30:15 ratio and reduced quantitative sudomotor axon reflex test sweat volume (59).

### Limitations

Further clinical studies are warranted and needed to evaluate whether there is a direct link between the increased STV_QT_ detected in the present study and increased risk for sudden cardiac death in patients with IGT, preferably in a large patient cohort.

## Conclusion

The present study is the first to show that short-term QT interval variability is higher in patients with IGT. The elevated temporal STV_QT_ and concomitant cardiac AN may serve as early indicators of the increased instability of cardiac repolarization and elevated risk for sudden cardiac death in patients with prediabetic states.

## Ethics Statement

The studies described here were carried out in accordance with the Declaration of Helsinki (2000) of the World Medical Association and were approved by the Scientific and Research Ethical Committee of the Medical Research Council at the Hungarian Ministry of Health (ETT-TUKEB), under ethical approval No. 4987-0/2010-1018EKU (338/PI/010). All subjects have given written informed consent of the study.

## Author Contributions

The authors listed below gave the following contributions: IB, AN, TV, LB, GÁ, PK, JP, AV, and CL had substantial contributions to the conception of the work and design of the paper; AO, SN, AK, ZP, RT, LB, and CL contributed to the measurements and analyses of data; AO, IB, PK, JP, AV, and CL drafted the paper or revised it critically for important intellectual content. All authors (AO, IB, SN, AK, ZP, RT, AN, TV, LB, GÁ, PK, JP, AV, and CL) have read and approved the final manuscript.

## Conflict of Interest Statement

The authors declare that the research was conducted in the absence of any commercial or financial relationships that could be construed as a potential conflict of interest.
